# Genomic alterations accompanying tumour evolution in colorectal cancer: tracking the differences between primary tumours and synchronous liver metastases by whole-exome sequencing

**DOI:** 10.1186/s12885-018-4639-4

**Published:** 2018-07-20

**Authors:** M. B. Mogensen, M. Rossing, O. Østrup, P. N. Larsen, P. J. Heiberg Engel, L. N. Jørgensen, E. V. Hogdall, J. Eriksen, P. Ibsen, P. Jess, M. Grauslund, H. J. Nielsen, F. C. Nielsen, B. Vainer, K. Osterlind

**Affiliations:** 1 Department of Oncology, Section 5073, Rigshospitalet, Copenhagen University Hospital, Blegdamsvej 9, 2100 Copenhagen, Denmark; 2 Center for Genomic Medicine, Rigshospitalet,Copenhagen University Hospital, 2100 Copenhagen, Denmark; 3 Department of Surgical Gastroenterology and Transplantation, Rigshospitalet,Copenhagen University Hospital, 2100 Copenhagen, Denmark; 4 Department of Pathology, Roskilde Hospital, Copenhagen University Hospital, 4000 Roskilde, Denmark; 5 Digestive Disease Center, Bispebjerg Hospital, Copenhagen University Hospital, 2400 Copenhagen, Denmark; 6 Department of Pathology, Herlev Hospital, Copenhagen University Hospital, 2730 Herlev, Denmark; 7 Department of Surgical Pathology, Zealand University Hospital, Slagelse Hospital, 4200 Slagelse, Denmark; 8 Department of Pathology, Hvidovre Hospital, Copenhagen University Hospital, Copenhagen, 2650 Hvidovre, Denmark; 9 Department of Surgical Gastroenterology, Roskilde Hospital, Copenhagen University Hospital, 4000 Roskilde, Denmark; 10 Department of Surgical Gastroenterology, Hvidovre Hospital, Copenhagen University Hospital, 2650 Hvidovre, Denmark; 11 Department of Pathology, Rigshospitalet,Copenhagen University Hospital, 2100 Copenhagen, Denmark

**Keywords:** Colorectal cancer, Concordance, Metastatic cancer, Heterogeneity

## Abstract

**Background:**

Colorectal cancer (CRC) patients with metastatic disease can become cured if neoadjuvant treatment can enable a resection. The search for predictive biomarkers is often performed on primary tumours tissue. In order to assess the effectiveness of tailored treatment in regard to the primary tumour the differences in the genomic profile needs to be clarified.

**Methods:**

Fresh-frozen tissue from primary tumours, synchronous liver metastases and adjacent normal liver was collected from 21 patients and analysed by whole-exome sequencing on the Illumina HiSeq 2500 platform. Gene variants designated as ‘damaging’ or ‘potentially damaging’ by Ingenuity software were used for the subsequent comparative analysis. BAM files were used as the input for the analysis of CNAs using NEXUS software.

**Results:**

Shared mutations between the primary tumours and the synchronous liver metastases varied from 50 to 96%. Mutations in *APC*, *KRAS*, *NRAS*, *TP53* or *BRAF* were concordant between the primary tumours and the metastases. Among the private mutations were well-known driver genes such as *PIK3CA* and *SMAD4*. The number of mutations was significantly higher in patients with right- compared to left-sided tumours (102 vs. 66, *p* = 0.004). Furthermore, right- compared to left-sided tumours had a significantly higher frequency of private mutations (*p* = 0.023). Similarly, CNAs differed between the primary tumours and the metastases. The difference was mostly comprised of numerical and segmental aberrations. However, novel CNAs were rarely observed in specific CRC-relevant genes.

**Conclusion:**

The examined primary colorectal tumours and synchronous liver metastases had multiple private mutations, indicating a high degree of inter-tumour heterogeneity in the individual patient. Moreover, the acquirement of novel CNAs from primary tumours to metastases substantiates the need for genomic profiling of metastases in order to tailor metastatic CRC therapies. As for the mutational status of the *KRAS*, *NRAS* and *BRAF* genes, no discordance was observed between the primary tumours and the metastases.

**Electronic supplementary material:**

The online version of this article (10.1186/s12885-018-4639-4) contains supplementary material, which is available to authorized users.

## Background

Colorectal cancer (CRC) is the third most common type of malignancy, accounting for 693,900 cancer-related deaths worldwide in 2012 [1]. Approximately 20% of patients with CRC present distant metastases at the time of diagnosis, most often located in the liver. The treatment of disseminated CRC has improved significantly during the last decade, including systemic as well as surgical treatment. This progress has enabled the cure of metastatic disease in cases where the metastases are resectable at diagnosis or after neoadjuvant chemotherapy [2]. Therefore, it is very important to select the best chemotherapy combination for neoadjuvant treatment of the individual patient. A resected primary tumour offers an accessible archetypal cancer tissue for the formulation of a genomic-guided treatment composition. The success of such a policy depends on whether the metastases display major differences in their genomic and thus proteomic profiles compared to those of the primary tumours [3, 4].

A diagnosis of CRC is in general based on biopsies from the primary tumour followed by resection; biopsies from metastases are rarely performed [5, 6]. Previous studies of CRC comparing mutations in the primary tumour and metastases from the same patient have focused on selected mutations in a few or a restricted panel of genes, i.e. mostly known cancer drivers such as *KRAS*, *BRAF* and *APC*. Mutations in the *APC* and *KRAS* genes appear early in tumour development and concordance between the primary tumour and metastases is seen in 88–100% of patients for *KRAS* mutations [7–11]. Due to genomic instability, it is reasonable to speculate that for many genes other than *KRAS* the genomic profile of metastases may show a greater deviation from the profile of the primary tumour. Therefore, to clarify how well the primary tumour reflects the metastases in CRC, in the present study we undertook whole exome sequencing for genomic profiling of fresh tissue from synchronous primary tumours and liver metastases.

## Methods

### Patients

Patients with colorectal liver metastases referred to Rigshospitalet (RH), Copenhagen University Hospital, during the period March 2012 to January 2014 were eligible for the study. To be eligible, the patient’s liver metastases had to be determined resectable at a multidisciplinary team conference and the patient’s primary tumour had to still be in situ or fresh-frozen tissue from a previously resected primary tumour, archived at the Danish CancerBiobank, had to be available [12]. Informed, written consent was obtained from all patients before inclusion in the study [12]. The inclusion criteria were a histologically confirmed adenocarcinoma, one or more synchronous, resectable liver metastases and an in situ primary tumour or tissue samples in the biobank. Patients who had received neoadjuvant chemotherapy could be included, but neoadjuvant radiotherapy was not allowed. The study protocol was approved by The Ethics Committee of the Capital Region of Denmark (H3–2011-150). The study was conducted in accordance with the Helsinki Declaration [13].

### Sampling and DNA extraction

Specimens were promptly collected at surgery, put on ice and transferred to the Pathology Department where sampling was performed immediately after arrival according to guidelines [12]. To allow sampling for the study, the tumour had to be large enough to ensure sufficient material for the standard diagnostic and staging pathologic examinations. Samples from the primary tumours were frozen to − 80 °C to ensure the DNA quality and a minor tumour fragment was formalin-fixed and paraffin embedded for microscopy by a pathologist. Only specimens with a tumour content of more than 70% were included in further analyses. The sampling and preparation of tissue from the liver resections were conducted in a similar manner. In addition to metastasis specimens, a piece of normal liver tissue was taken, plus a formalin-fixed specimen for microscopy. DNA was extracted from the fresh-frozen tissue using the NucleoSpin Tissue Kit (Macherey-Nagel GmbH & Co. KG, Germany) according to the manufacturer’s instructions, with the added application of a tissue lyzer in the lysis step. DNA quantity was assessed by Agilent’s Bioanalyzer (USA).

### Whole-exome sequencing and data processing

Enrichment of the exome was performed using Nimblegen Exomes (Nimblegen SeqCap EZ Human Exome Library v3.0) based on 64 Mb capture of coding region without 5′ and 3’ UTR (Nimblegen, USA). Sequencing was performed on the Illumina HiSeq 2500 platform. In brief, 1 μg of genomic DNA was fractionated on a Covaris S2 to an average size of 200 bp. Trimming, 3′ adenylation and ligation of Illumina TruSeq DNA adaptors were performed on an SPRI-TE Nucleic Acid Extractor using SPRIworks Fragment Library Cartridges I (Beckman Coulter, USA) with a size selection of 200–400 bp. Following sequencing, paired FASTQ files were generated using Illumina CASAVA-1.8.2 software and imported into CLC Genomics Workbench (Qiagen, Germany) for further processing. The reads were aligned to the reference haploid human genome sequence (Genome Reference Consortium human genome build 37, human genome 19 (*hg19*)). A further local realignment was performed to improve the alignment of individual reads in the presence of insertions and deletions relative to the reference.

From each patient three samples were sequenced and filtered: primary tumour, liver metastasis and normal liver tissue. Single nucleotide variants (SNV) from normal liver tissue were extracted from the gene variants called from the primary tumour and liver metastases to exclude germline variants. By the use of CLC Genomic Workbench SNV were then filtered according to a minimum count (> 5), a maximum count in control (< 10), coverage (> 9) and mutation allele frequencies along with a forward-backward reading demand (> 0.2). After filtering, variants were exported as VCF files to enable Ingenuity Variant Analysis (Qiagen, Germany) for further filtering based on predicting of the damaging effect of variants. Classification of Ingenuity is supported by data from other in silico prediction algorithms such as SIFT, PolyPhen and COSMIC [14–16]. Only variants described with lower than 1% frequency in the 1000 Genomes Project or National Heart, Lung and Blood Institute Exome Sequencing Project were included, because variant genes present in more than 1% of the population are thought to be common gene variants and not associated with a disease. In addition, the Ingenuity Variant Analysis was performed to keep only variants being associated with a loss of function, frameshift, in-frame, stop codon or missense and in addition being attributed: ‘pathogenic’ or ‘likely pathogenic’. CLC Genomics Workbench was subsequently used for manual visual control and exclusion of false positive variants. By visual control of called variants, the same positions were identified in the primary tumour, metastases and normal liver tissue, ensuring that variants called private were not caused by the filtering procedure when the frequency was low and thus falsely attributed as ‘private’. The visual control also ensured that variants called ‘private’ were not classified as ‘private’ because the other site was not uncovered. Variants called ‘shared’ meant not only a mutation in the same gene but the exact same mutation in both the primary tumour and the metastasis. The gene variants were classified according to the SIFT system [16] which sorts intolerant from tolerant substitutions based on a prediction of potential substitution effects on the resulting protein function, being divided into ‘tolerated’, ‘damaging’ or ‘activating’. The SIFT classification was called by the Ingenuity software. Driver genes, based on a prior study reporting a list of high confidence driver genes (291 driver genes) were identified among our variants, but not used as a filter [17]. To estimate the amount of mutations that was only subclonal in the primary tumour but had a high frequency in the metastases, an increase in allel frequency of > 30 from the primary tumour to the metastases was selected (∆30%).

Genes identified as private to the metastases were included in a network analysis using the Ingenuity Pathway Analysis (IPA) programme [18]. The IPA determination of whether pathways are significantly linked to the gene set under investigation is based on Fisher’s exact test. Further, the gene sets were investigated by the Gene Set Enrichment Analysis (GSEA; http://software.broadinstitute.org/gsea/msigdb) which associates gene sets with phenotypes using a predefined collection of data sets (4725 gene sets in the collection) [19].

BAM files generated from the exome sequencing were used as the input for the derivation of genomic profiles for analyses of CNAs using BioDiscovery software, NEXUS 8.0 (BioDiscovery, USA). Sixteen BAM files from adjacent normal liver samples were used to create a study reference according to the software instructions. BAM files from primary tumours and metastases were subsequently analysed by standard recommendations of the manufacturer. The quality of sequenced data from five patients were suboptimal for CNA analysis, thus these patients were excluded from further CNA analyses. Raw CNAs as numerical (involving the whole chromosome) and segmental (involving part of the chromosome in size at least of one chromosomal band) were manually assessed. Local alterations in individual genes, selected due to a conventionally accepted relevance for CRC, were also analysed [20].

### Validation by gene panel

Tissue from primary tumours were used for validation of selected hotspot mutations identified by WES using the AmpliSeq Colon Lung Cancer Panel version 2 (Thermo Fisher Scientific, USA) comprising hotspot regions of 22 onco- and tumour suppressor genes. Eighteen primary samples were included for validation analysis.

DNA was extracted from a 5 μm thick section of formalin-fixed paraffin-embedded tumour sample using QIAamp DNA Mini Kit (Qiagen) according to manufacturs instructions and quantified with a Qubit Photometer. Ten ng of DNA was used for library preparation using the Ion AmpliSeq Library Kit 2.0 and the AmpliSeq Colon Lung Cancer Panel version 2 (Life Technologies). Sequencing libraries were quantified by the Ion Library TaqMan ™ Quantification Kit and manually loaded on Ion 314 or 316 sequencing chips and sequenced > 500× in coverage by the Ion Torrent Personal Genome Machine (Thermo Fisher Scientific). Data analysis was carried out using the Torrent Suite browser version 4.4 (Thermo Fisher Scientific) and variants manually inspected in Integrative Genomics Viewer (Broad Institute, USA).

### Statistical analysis

All statistical analyses were performed using SPSS software version 19 (IBM, USA). The correlation between the number of mutations and the location of the primary tumour, between the number of mutations and neoadjuvant treatment or not, and between percentages of shared mutations and the localization of the primary tumour was analysed by the Mann-Whitney U-test. The Kruskal-Wallis test was used to determine whether the distribution of shared mutations depended on the sequential order of resections. A generalized linear model was constructed to examine whether the sequential order of the two resections, chemotherapy (if given between the two operations), and the localization of the primary tumour influenced the number of private mutations. Tests with a *p*-value less than 0.05 were considered statistically significant.

## Results

### Patient characteristics

**Fig. 1:**
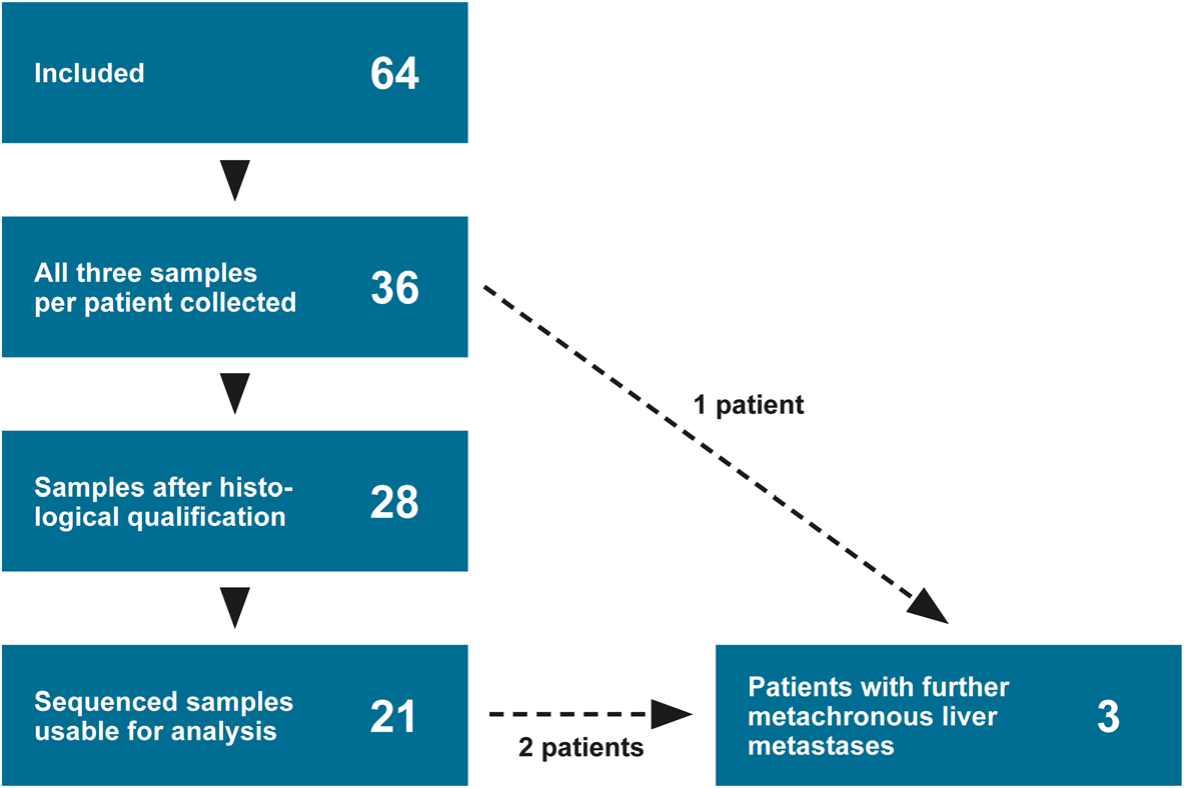
CONSORT flow diagram of patients reviewed for inclusion in the study

Sixty-four consecutive patients were included in the study. Samples from all three sites were successfully obtained from 36 patients (Fig. 1). The remaining patients were excluded due to cancellation of planned surgery, acute surgery or the resected primary tumour was too small to allow for tissue collection for the study. Eight samples were excluded after the histological evaluation, and an additional seven samples failed the quality controls after sequencing. Thus, 21 patients remained, resulting in a total of 63 samples. Three patients had additional metachronous metastases (removed at a subsequent liver resection) available for analysis (Fig. 1). The characteristics of the 21 patients are listed in Table 1. All tumours were microsatellite stable, i.e. had normal expression of the four mismatch repair (MMR) proteins MLH1, MSH2, MSH6 and PMS2. Immunohistochemistry examination of these four proteins is standard procedure for all resected CRC patients in Denmark.

**Table 1:** Patient characteristics

Characteristics	*n* = 21
Age	Median 68 (38–84)
Gender	
Male	12 (57%)
Female	9 (43%)
Primary tumour location	
Right	9 (43%)
Left	12 (57%)
Resection	
Concurrent	5 (24%)
Subsequent – liver first	5 (24%)
Subsequent – primary first	11 (52%)
Time between resections	Median 36 days
Treatment	
Chemonaïve	11 (52%)
Both tumours	7 (33%)
Only metastasis	2 (10%)
Only primary	1 (5%)
Neoadjuvant treatment	
CAPOX	2 (10%)
CAPOX + bevacizumab	2 (10%)
FOLFIRI + cetuximab	1 (5%)
FOLFOX + cetuximab	2 (10%)
Between resections	
CAPOX	2 (10%)
FOLFOX	1 (5%)
Number metastases	Median 2 (1–5)
Microsatellite stability	21 (100%)
Tumour stage	
Stage IV	21 (100%)
Tumour differentiation	
Low	2 (10%)
High	19 (90%)

*CAPOX* denotes capecitabine and oxaliplatin, *FOLFIRI* signifies 5-fluorouracil and irinotecan; and *FOLFOX* indicates 5-fluorouracil and oxaliplatin

### Classification of mutations in primary tumours and metastases

An average of 180,285 gene variants was identified per sample by the initial workflow. Filtering decreased the number of variants to a mean of 470 per sample eligible for manual visual identification, resulting in an average of 73 (41–142) mutations per patient including shared and private mutations from both sites. A mean of 92% (72–100%) of variants designated as shared were identified in the filtering process in both primary tumour and metastasis, the remaining 8% were identified at the manual visual control of the paired sites.

Transitions (TI; T↔C; G↔A) were identified in 62.0% and transversions (TV; T↔A; G↔C; A↔C; T↔G) in 31.2%, giving a TI/TV ratio of 2.0 (range 1.0–3.8). There was no difference in the TI/TV ratios between primary tumours and metastases (*p* = 0.8).

**Fig. 2:**
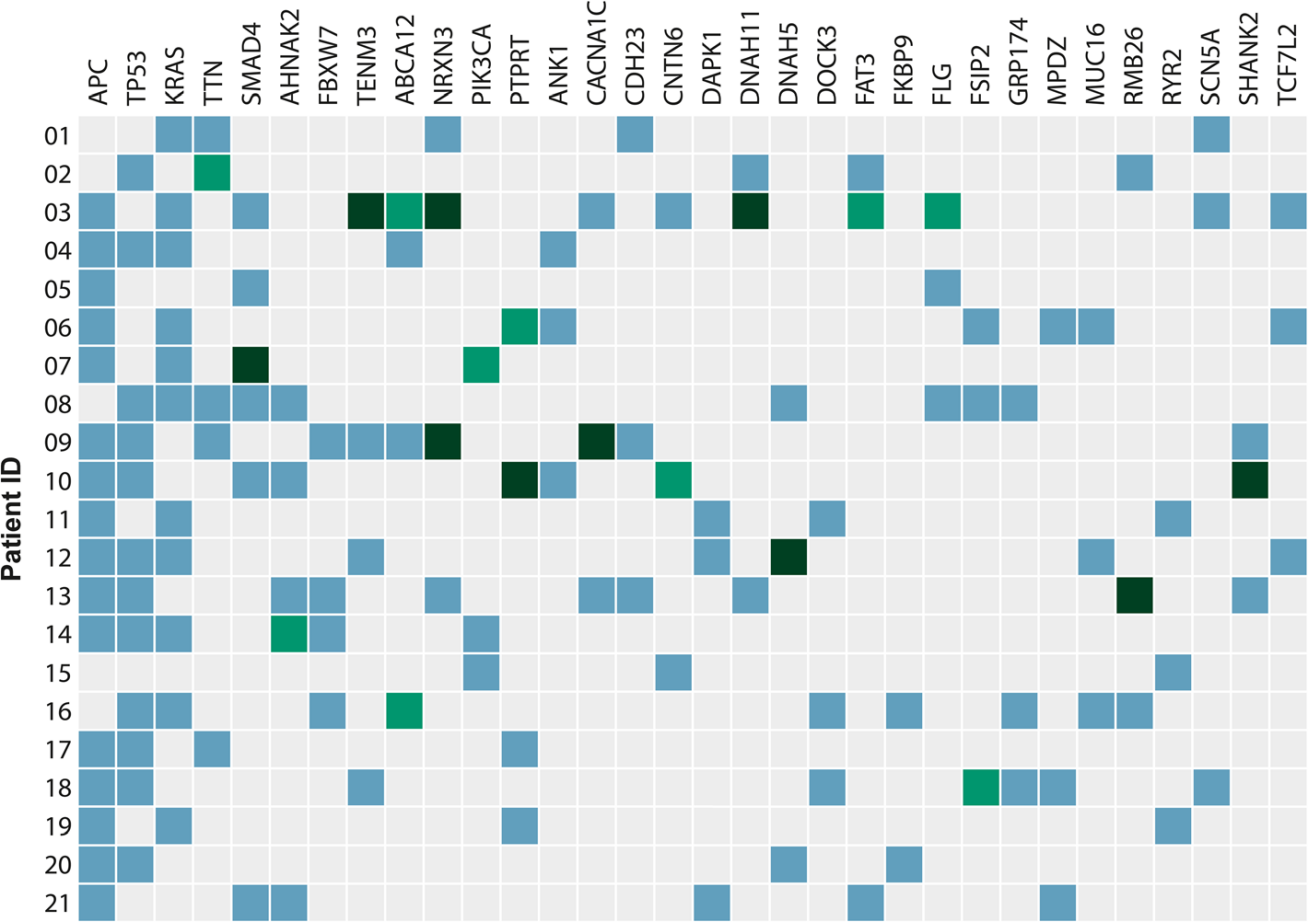
The genes in which mutations were most highly represented are illustrated. Blue-filled box denotes mutation shared between the primary tumour and the metastasis, light green-filled box signifies mutation private to the primary tumour and dark green-filled box indicates mutation private to the metastasis. Shared mutation refers to the exact same mutation and not just a mutation located in the same gene

In total, 1674 variants/mutations located in 1435 different genes were identified in the 21 paired tumour samples, with *APC* (76%), *TP53* (57%) and *KRAS* (52%) being the genes with the highest mutation rates. The most frequently mutated genes in our cohort are presented in Fig. 2. The average coverage reported by the CLC workflow per sample had a mean of 47.2 in primary tumours and 43.8 in metastases. There was no difference in the coverage of the primary tumours and their paired metastases (*p* = 0.25). The gene variants identified had an average sequence coverage of 77.3.

**Fig. 3:**
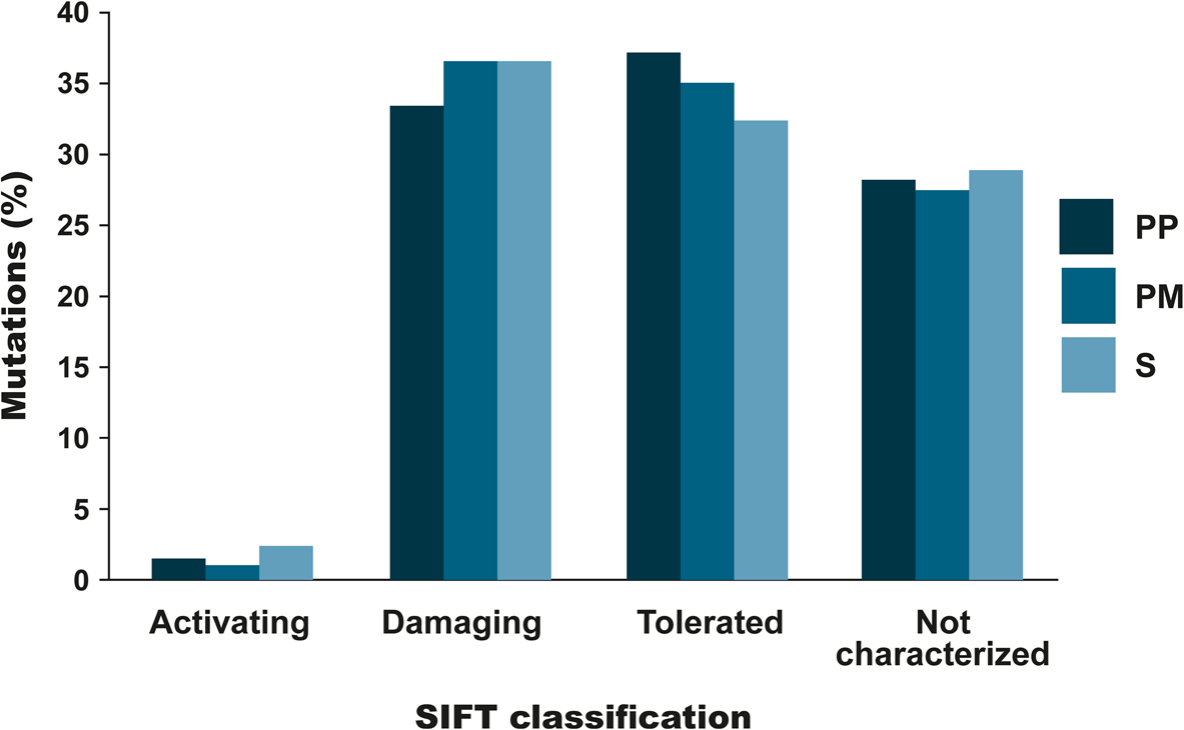
The characterization of mutations according to the SIFT classification divided into private mutations to the primary tumour (PP), private mutations to the metastases (PM) and shared mutations (S). A nearly identical distribution of activating and damaging mutations relative to the private and shared mutations was revealed

The identified gene variants were subsequently assigned a status of either shared mutation (S) (common between the primary tumour and metastases), private to the primary tumour (PP) or private to the metastases (PM). Interestingly, mutations with a different status (i.e. shared vs. private) had similar SIFT classification patterns (Fig. 3), with nearly the same frequency of activating (S 2.2%, PM 1.0%, PP 1.5%) and damaging mutations (S 36.6%, PM 36.6%, PP 33.5%). Searching for known driver genes a total of 117 called variants were identified in a driver gene, ranging from 3 to 11 among the patients (median 5). 15.4% of these variants in driver genes were private.

Genes with a private mutation in the metastasis were submitted to IPA. These genes were found to be involved in processes such as cell movement, cell-to-cell signalling and interaction, cell death and survival, and cell morphology (IPA; *p* < 0.05). Assuming that mutations may have an effect on the expression of the mutated gene, GSEA analysis was undertaken and disclosed that private mutations in metastases resembled signatures found in a subset of patients with nasopharyngeal carcinoma (GSEA; ODD_NASOPHARYNGEAL_CARCINOMA_UP; FDR q-value 8.33E-5).

### Specificity of private mutations

**Fig. 4:**
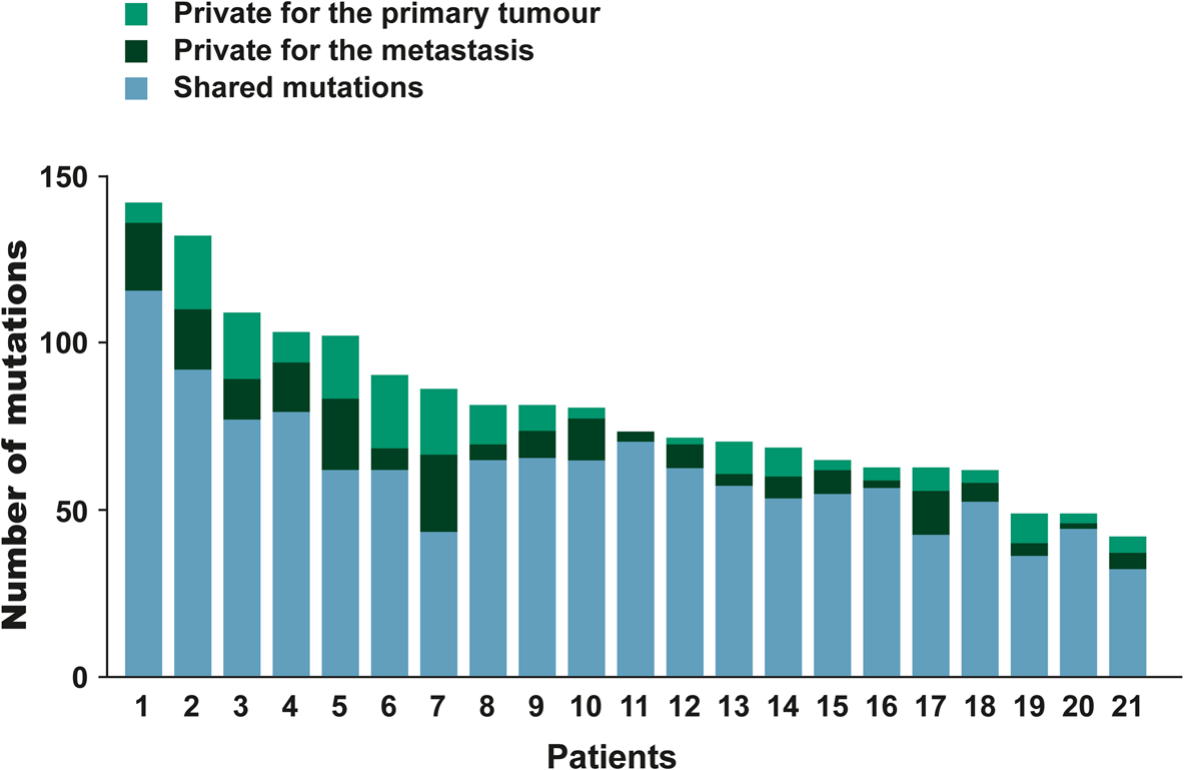
Histogram showing the mutational number according to each patient divided into shared and private mutations. Blue represents shared mutations, light green signifies mutations private to the primary tumour and dark green indicates mutations private to the metastases

Shared mutations were dominant with a median of 78% ranging from 50 to 96%. About one tenth of all mutations identified were private in the primary tumour (12%) as well as in the metastases (12%). Hence, some mutations from the primary tumour were not identified in the metastases or had evolved in the primary tumour after metastatic spread and some evolved in the metastases. The relatively high proportion of shared mutations indicates that the primary profile is largely maintained during disease progression. The occurrence of shared versus private mutations is summarised in Fig. 4.

Concordance between mutations in the primary tumour and the synchronous liver metastasis was seen in *APC*, *KRAS*, *NRAS*, *TP53* and *BRAF* genes (100%), while discordance among those with a mutation was seen in, amongst others, *PIK3CA* (50%), *AHNAK2* (20%), *SMAD4* (17%), *BCLAF1* (50%), and *ARHGAP32* (100%).

**Fig. 5:**
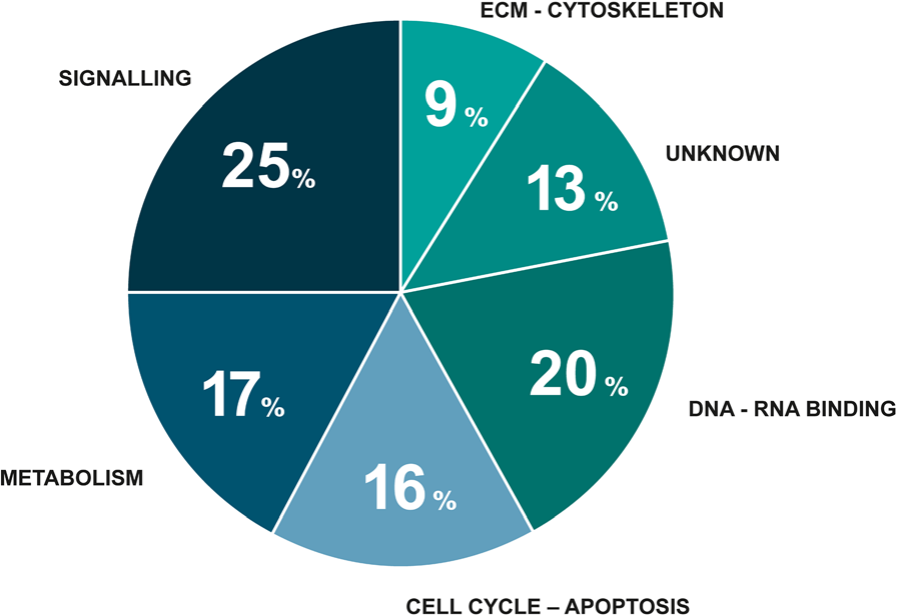
Functional categories of the genes private to the metastasis and categorized according to SIFT as damaging or activating. In total, 76 genes were included. The GO annotations in the Ingenuity software were used for functional assessment. The genes were grouped according to the functional categories: DNA/RNA binding, cell cycle – apoptosis, metabolism, signalling, ECM – cytoskeleton, and unknown

In total, 196 private mutations were identified in the liver metastases and 194 private mutations were found in the primary tumours of the 21 patients (Additional file 1: Table S1). All 390 private mutations were unique, i.e. none were seen as private in two or more patients. However, 74 of the private mutations were also seen in other mutations as shared. Figure 5 shows the functions of these genes distributed into six categories: DNA or RNA binding, cell cycle and apoptosis, metabolism, signalling, extracellular matrix or cytoskeleton and unknown, with 25% categorized as important for signalling and 20% involved in DNA or RNA binding.

Interestingly, one patient had two primary T3 tumours located in the ascending colon with only 3 cm in between. The tumours harboured 45 and 64 mutations, respectively. None of the mutations found in the primary tumours were shared, reflecting different origins of these lesions. Of note, two different activating *KRAS* mutations (*KRAS* c.38G > A, *KRAS* c.436G > A) were identified in these tumours. The examined liver metastasis in this patient had 79% shared mutations with the primary tumour harbouring the *KRAS* c.38G > A mutation, while no mutations were shared with the other primary tumour.

With the reservation of possible normal tissue contamination influencing the frequencies, a median of two mutations per primary tumour/metastases pair proved to have an increase of more than 30 in allel frequency. Noteworthy, *TP53* mutations (c.944C > T; c.794 T > C; c.817C > T in three patients (36% → 78%; 11 → 47%; 41 → 73%), a *SMAD4* (c.1067C > T) in one patient (24% → 73%) and an *AKT* (c.49G > A) mutation in one patient (37% → 84%). A total of 35 of 36 mutations identified by WES was validated by the hot spot panel i.e. only one mutation (*KRAS*, c.38G > A) was not identified by the hot spot panel.

### Metachronous liver metastasis

**Fig. 6:**
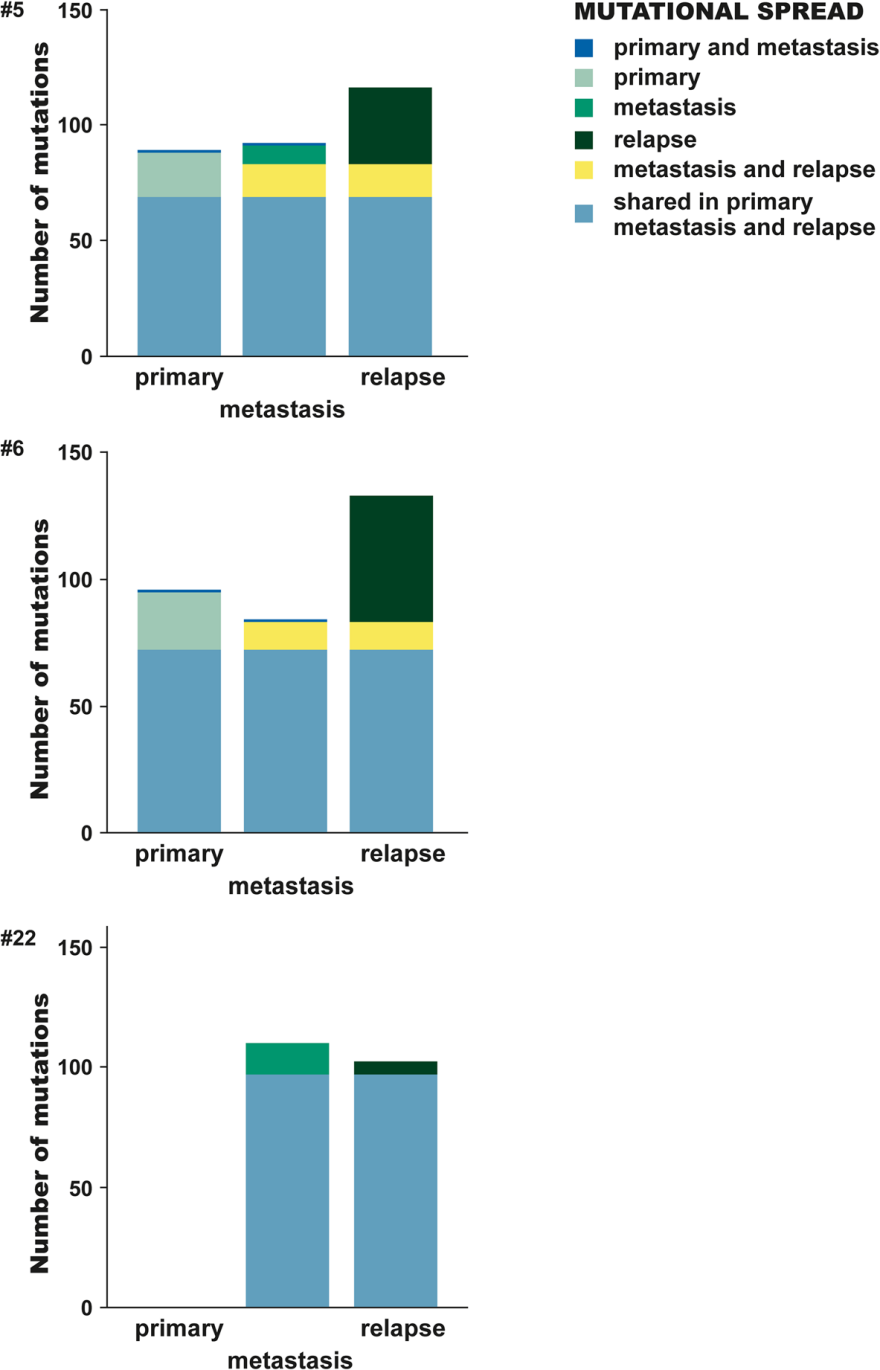
Mutations located in the primary tumour, synchronous liver metastasis and metachronous liver metastasis. Patient(#) 5 and 6 had all three sites successful sequenced and analysed, #22 had a primary tumour with insufficient residual tumour content and therefore not analysed. The bar of each site (primary tumour, synchronous metastasis, relapse) are coloured according to the identified variants being shared with another site or private

Three patients operated for a subsequent relapse had an additional, metachronous liver metastasis sequenced, patients #5, 6 and 22 as corresponding to patient ID in Figs. 2 and 4. The distributions of mutations in the primary tumour and in the syn- and metachronous liver metastases, respectively, are shown in Fig. 6. The specimen from the primary tumour of patient #22 had a high degree of normal tissue and was therefore omitted, leaving only the synchronous and metachronous liver metastases for comparison. Among all mutations identified in #5 and #6, 48 and 46% was seen as shared between all three sites, respectively, and 23% (#5) and 32% (#6) of identified variants proved to be private in the metachronous metastases. As for the two primary tumours, 13% (#5) and 15% (#6) of all variants identified in the patient were private mutations, i.e. mutations that may have evolved after the metastatic spread or been lost during the process of dissemination. Private mutations in the three metachronous metastases were, amongst others, *SORBS1*, *AR*, *CNTN1*, *TIMP3*, *PLK1*, *SIRT7*, *DNAH5* and *BRCA2*.

### Comparison of mutational burden according to primary tumour location

**Fig. 7:**
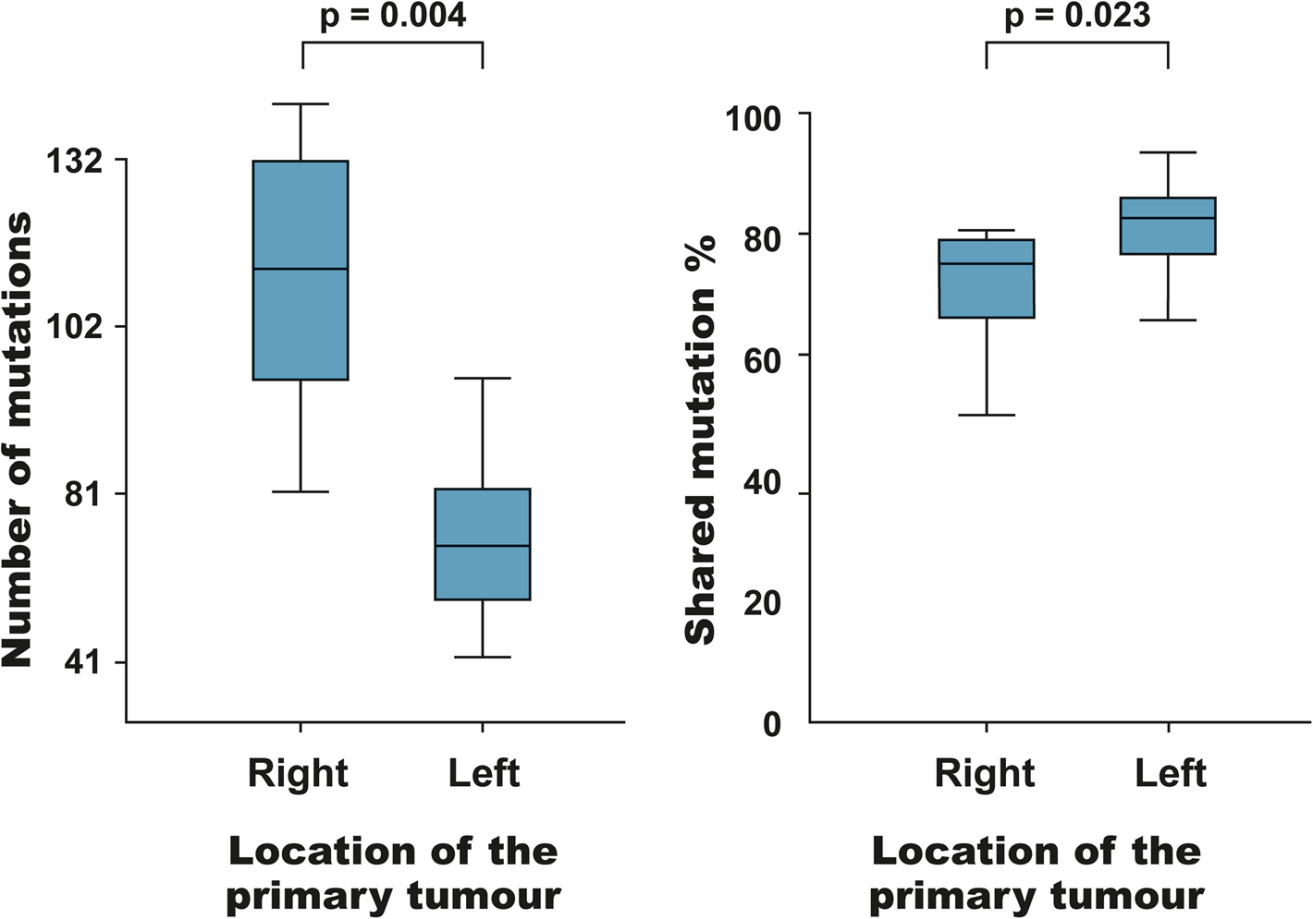
Left panel: Box plot illustrating the number of mutations according to the location of the primary tumour. A significantly higher number of mutations (mutational burden) was observed in patients with a right-sided tumour (*p* = 0.004). Right panel: Box plot illustrating the percentage of shared mutations according to the location of the primary tumour. A significantly higher degree of shared variants was seen in patients with a left-sided tumour (*p* = 0.023)

Right- and left-sided bowel tumours are evolutionarily different, deriving from mid- and hindgut, respectively. This may explain the different mutation patterns and biological behaviour comparing right- with left-sided bowel cancers [21, 22]. Therefore, the numbers of mutations per patient (S + PP + PM) in left- versus right-sided primary tumours were compared. The number of mutations (i.e the mutational burden) was significantly higher in the right-sided tumours (median of 102 vs. 66, *p* = 0.004; Fig. 7). Comparing only mutations present in the primary tumour (S + PP) of left- versus right-sided, a significant difference was still present (*p* = 0.021). Moreover, patients with right-sided tumours had significantly fewer shared mutations (75% vs. 82%, *p* = 0.023; Fig. 7).

### Mutational profile and chemotherapy

The number of mutations was not related to whether the patient had received neoadjuvant treatment or was chemonaïve (*p* = 0.4). Similarly, chemotherapy between the first and second resection had no impact on the percentages of shared mutations (*p* = 1.0), neither did the sequential order of resection history: liver before bowel or vice versa (*p* = 0.3). No confounding was observed from ‘chemotherapy between bowel and liver surgery’ or ‘sequential order of operations’ (generalized linear model). However, this outcome of our analyses must be interpreted cautiously since the study was not powered to answer this question. Few patients received chemotherapy between their resections and number of cycles varied from patient to patient. The time spans between resection of the synchronous and the metachronous liver metastasis were 14, 18 and 3 months, respectively. At this point, 15, 8 and 1 cycle (s) of chemotherapy had been administered.

### Copy number aberrations

**Fig. 8:**
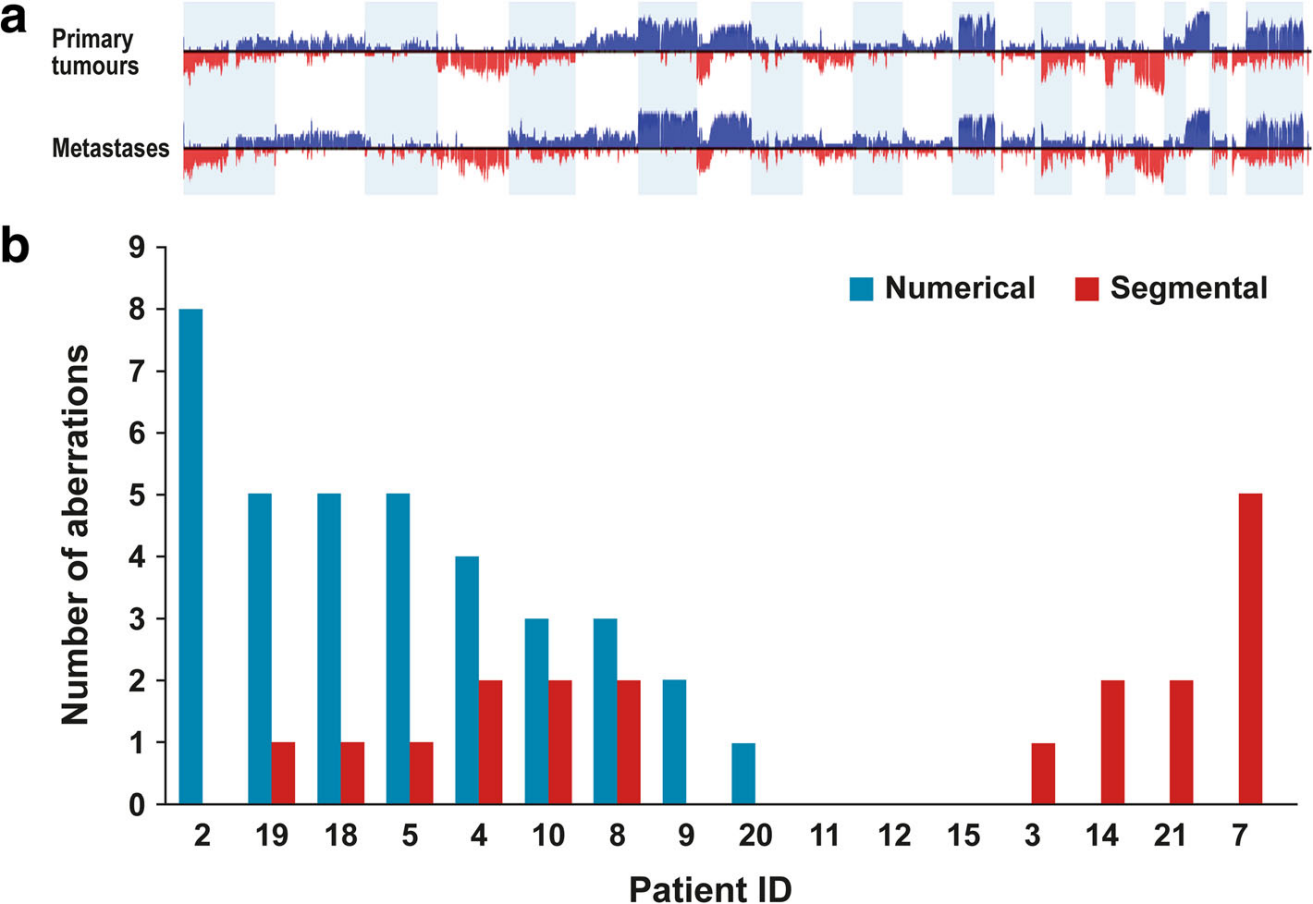
**a**) Frequency plot of copy number aberrations in primary tumour (upper plot) and synchronous liver metastases (lower plot). Red indicates deletions and blue indicates copy number gains. **b**) Histogram illustrating of copy number aberrations – numerical as well as segmental - in 16 pairs of primary tumour and synchronous liver metastasis. Patients on the x-axis are numbered as in Figs. 3 and 5

We further used the sequence data to derive CNA profiles. Adjacent normal liver tissue samples were used as a reference. Numerical aberrations occurred to a higher extent in chromosomes 7, 8, 13, 18 and 20, and segmental aberrations were more common in chromosomes 1, 5, 8 and 18 (Additional file 2: Table S2). Allelic frequencies for primary tumour and metastases are summarized in Fig. 8a. Comparing numerical and segmental aberrations in the primary tumour versus metastasis, every pair differed in at least one of these raw chromosomal aberrations (Fig. 8b). Interestingly, there was a trend that metastases expressing a high number of novel numerical aberrations had fewer segmental aberrations. Conversely metastases with many novel segmental aberrations had fewer numerical aberrations (Fig. 8b).

Local CNAs of individual genes, selected because of their relevance in CRC, showed almost no differences between the primary tumour and metastasis (Additional file 3: Table S3). This is in agreement with our observations at the mutational level, where driver events remained unaltered during tumour progression. *SMAD2*, *SMAD4*, *TP53* and *AURKA* were affected by CNAs in a high proportion of the patients, i.e. in 11, 11, 8 and 6 out of 16 patients, respectively.

Damaging private mutations (according to the SIFT classification) identified in the metastases were investigated to determine whether CNAs accompanied the mutation. Twelve of the mutated genes showed CNAs, nine with a loss of heterogeneity (Additional file 4: Table S4).

## Discussion

Several studies of various cancer types have suggested that the mutation status of the primary tumour has limitations as an indicator for the selection of treatment of the metastatic disease. Studies of breast cancer [23, 24] and central nervous system tumours [25] have provided evidence supporting the view that primary lesions and metastases are individual genomic entities; however, with regard to CRC, solid evidence still remains to be presented. The present study compared the genomic profiles of paired primary tumours and synchronous liver metastases from CRC patients and found from 50 to 96% concordance between the primary tumour and the metastasis in the investigated patients, i.e. these data are not in support of the view of genomic entities.

The prevailing mutations were *APC*, *KRAS* and *TP53*, which is in accordance with data reported by The Cancer Genome Atlas Network [26]. In addition, a total of 1435 different genes were found to be mutated, illustrating the complexity and therefore the importance of defining specific profiles if clinically applicable conclusions are to be possible. Furthermore, only a few mutations were similar among the 21 investigated patients, indicating that a large group of patients has to be examined to determine whether a correlation between a new specific mutations and a specific treatment do exist. The example of the patient with two closely located primary right-sided tumours having completely different genomic profiles emphasises the importance of mutational analysis of all primary tumours of a patient and of the metastasis if it is relevant for the treatment strategy.

The present results add to the body of evidence that microsatellite stable CRC has an average mutation rate of 60 [27]. The functional impact of mutations was further examined using in silico modelling by applying the SIFT classification. According to SIFT, 38.2% of the mutations were found to be either damaging or activating. Although in general in silico analysis has a relatively low specificity and high sensitivity making direct interpretation difficult in a clinical context, we found SIFT to be supportive for comparisons of the composition of gene variants. Our analyses revealed similar distributions of activating, damaging and tolerated mutations within the group of private mutations compared to the group of shared mutations, suggesting a lack of selectivity in the occurrence of private mutations. Most of the identified variants are probably passenger mutations, fewer expected to be in driver genes. The number of known cancer driver genes is still limited but growing with increasing knowledge about the function of cancer specific genes. The majority of the observed mutation variants in the cancer driver genes in our set of genes were present in both primary tumour and metastasis. It has previously been implied that at least three rate-limiting mutations are required to develop late-stage CRC [28]. Based on the observations that polyps with mutations in the *KRAS* gene do not develop to cancer and that hyperplastic polyps do not carry a mutation in the *APC* gene, it seems reasonable to speculate that the first rate-limiting step in the development of CRC are mutations in the APC pathway [29]. This theory is supported by the fact that small adenomas, advanced adenomas and carcinomas have almost the same frequency of mutations in the *APC* gene [30], and may explain why we saw all mutations in the *APC* gene as shared. Studies comparing the primary tumour with metastases are few in number and have mostly been performed on a panel of selected genes, with a high degree of concordance being reported [9, 31–33]. It appears that investigations focusing on a few genes result in a high concordance but expanding the panel of genes to include less common ones leads to a higher degree of discordance. We found a concordance of 50 to 96% of the mutational profile of the primary tumour compared to synchronous liver metastases. The number of private mutations may reflect events during the time from metastatic seeding to settling and metastatic growth in the liver. If the metastatic spread occurs early in the tumour development, a higher degree of diversity would be expected. Further, it is acknowledged that primary tumours can be very heterogeneous. This can be explained by the branched evolution, where subclones grow side-by-side progressing simultaneously and new subclones appear as new mutations develop. The number of different mutations between two cell samples is supposed to reflect the number of generations passed from the origin in a common mother cell. Several studies have shown a high degree of heterogeneity in primary tumours, not present to the same degree in metastases [34]. Sampling from another clone in the primary than the one which gave rise to the liver metastasis may lead to more private mutations in both. A study including several tumour types investigated tumour phylogenetic, and found a paraphyletic growth pattern in metastases indicating that there is no linear-specific event required to propagate metastasizing. This is in line with our finding of high inter-patient diversity, and of early driver genes seen in several patients. Zhao et al. further described drivers as KRAS and TP53 as early, PIK3CA and KMT2D midway events, and ALK and KMT2C to be late driver events [35].

The level of sequencing depth is important for the identified frequency of private mutations. It cannot be excluded that a few private mutations might not be private if the sequencing depths had been even higher; however, as private mutations were manually identified by the visual inspection of mapped genes we believe that it can only be very few. Furthermore, the average sequencing depth was not different between the primary tumour and the metastases.

High concordance was seen in the hot spot panel validating shared as well as some private mutations. A single *KRAS* mutation was not confirmed in the validation analysis, most likely due to heterogeneity in the primary tumour.

An *NRAS* mutation was found in one and *KRAS* mutations were found in 11 of 21 patients in this series and all were common. This is in accordance with previous findings [8–10] and supports the general praxis of analysing the *RAS* status in whatever tissue is available, primary tumour or metastasis. In general, studies on predictive factors relate likelihood of response to biomarker status of the primary tumour [36, 37]. This practice, however, may miss possible correlations due to a mutational status based on the primary tumour rather than the metastases. We found from 4 to 50% private mutations, meaning that if these patients were included in a biomarker study there would be a substantial risk of relating outcome to gene mutations being private to one site. Most of the identified variants are though assumed to be passenger mutations. Among identified variants in driver genes only few were private. As an example, the gene *PIK3CA* showed a mutation in the primary tumour but not in the metastases. A mutation in this gene may have an impact on the sensitivity to PI3K/AKT/mTOR inhibitors [38, 39]. With the exception of mutations in the *APC* and *KRAS* genes, it is not possible to point out genes which rarely occur as private mutations. In this study, we found the well-known driver genes *MAPK1*, *PIK3CA* and *SMAD4* to be private to either the primary tumour or the metastasis. Even though a mutation is present in a driver gene and might have an important role in the tumourigenesis it will not necessarily be a potential biomarker. In our current clinic setting only the *KRAS, NRAS* and *BRAF* status have importance for the treatment strategy but hopefully more will be identified. In general, predictive genetic biomarkers should be tested on tissue from the metastases that are the target for treatment.

A few of the found private mutations were categorised as activating and so these changes will most likely elicit a change in the cell. Mutations categorised as damaging are more questionable regarding whether they actually have an influence on the tumour. Since it is not possible to directly extrapolate the frequency of a found mutation in the whole-exome sequencing data, one can only guess whether the mutation is present on one or two alleles and in all the samples. Our analysis was therefore extended to include a copy number variant analysis to determine whether some of the private mutations in the metastases were coupled with copy number aberrations. Twelve of the identified private mutations in the metastases were shown to have a copy number aberration at their location as well, thus increasing the likelihood that the functions of the genes are affected. In this study, copy number changes were most prominent in chromosomes 1, 5, 7, 8, 13, 18 and 20, which is similar to the findings of other studies [40, 41]. Particular aberrations in chromosomes 8 and 20 have been reported to be correlated to tumour progression.

Right- and left-sided bowel tumours derive embryologically from the mid- and hindgut, respectively [42]. We found a higher number of mutations in right-sided tumours compared to left-sided ones. The difference could not be related to mismatch repair insufficiency since all tumours in this study were microsatellite stable. Furthermore, we found a lower degree of concordance between the primary tumour and liver metastases in patients with right-sided tumours. This is most likely a basic biological characteristic rather than a diagnostic-delay characteristic for right-sided cancers where the tumour tends to lead to symptoms considerably later than left-sided tumours [43].

## Conclusion

In this study of CRC considerable concordance was seen between mutations in the primary tumour and synchronous liver metastases, although both harbour many private mutations. Larger, prospective investigations are needed to clarify the therapeutic and prognostic implications of private mutations. Regarding *K*- and *NRAS* status, concordance is normal, supporting the current praxis to use whatever tissue is available. The mutational profiles of primary tumours may prove insufficient in a future where novel drugs might target mutations which frequently occur privately. However, our data do not point out gene mutations that are rarely found to be common.

## Additional files

Additional file 1: **Table S1.** Private mutations. Abbreviations: SIFT (sort intolerant from tolerant), PP (mutation private to the primary tumour), PM (mutation private to the metastasis). (XLSX 50 kb)

Additional file 2: **Table S2.** Identified numerical and segmental aberations in chromosomes in the primary tumour and metastases. (PPTX 53 kb)

Additional file 3: **Table S3.** Private copy number abberations are colored blue. Differences in copy number abberations are illustrated with / showing status in primary/metastasis. Abbreviations: LOH (loss of heterogeneity), D (deletion), A (amplification). (XLSX 14 kb)

Additional file 4: **Table S4.** Damaging private mutations (SIFT) identified to determine whether CNAs accompanied the mutation. (PPTX 47 kb)

## Abbreviations

CNA: Copy number aberrations
CRC: Colorectal cancer
DNA: Deoxyribonucleic acid
GSEA: Gene set enrichment analysis
IPA: Ingenuity pathway analysis
MMR: Mismatch repair
PM: Private to the metastases
PP: Private to the primary
RH: Rigshospitalet
RNA: Ribonucleic acid
S: Shared mutations
TI: Transitions
TV: Transversions
