# Supplementary material for: Genomic alterations accompanying tumour evolution in colorectal cancer: tracking the differences between primary tumours and synchronous liver metastases by whole-exome sequencing
Source: BMC Cancer. 2018 Jul 20;18:752. doi: 10.1186/s12885-018-4639-4 (PMC6053835; doi:10.1186/s12885-018-4639-4)
Supplement: Supplementary file 2 — Table S2. Identified numerical and segmental aberations in chromosomes in the primary tumour and metastases. (PPTX 53 kb) [file 12885_2018_4639_MOESM2_ESM.pptx]

## Slide 1
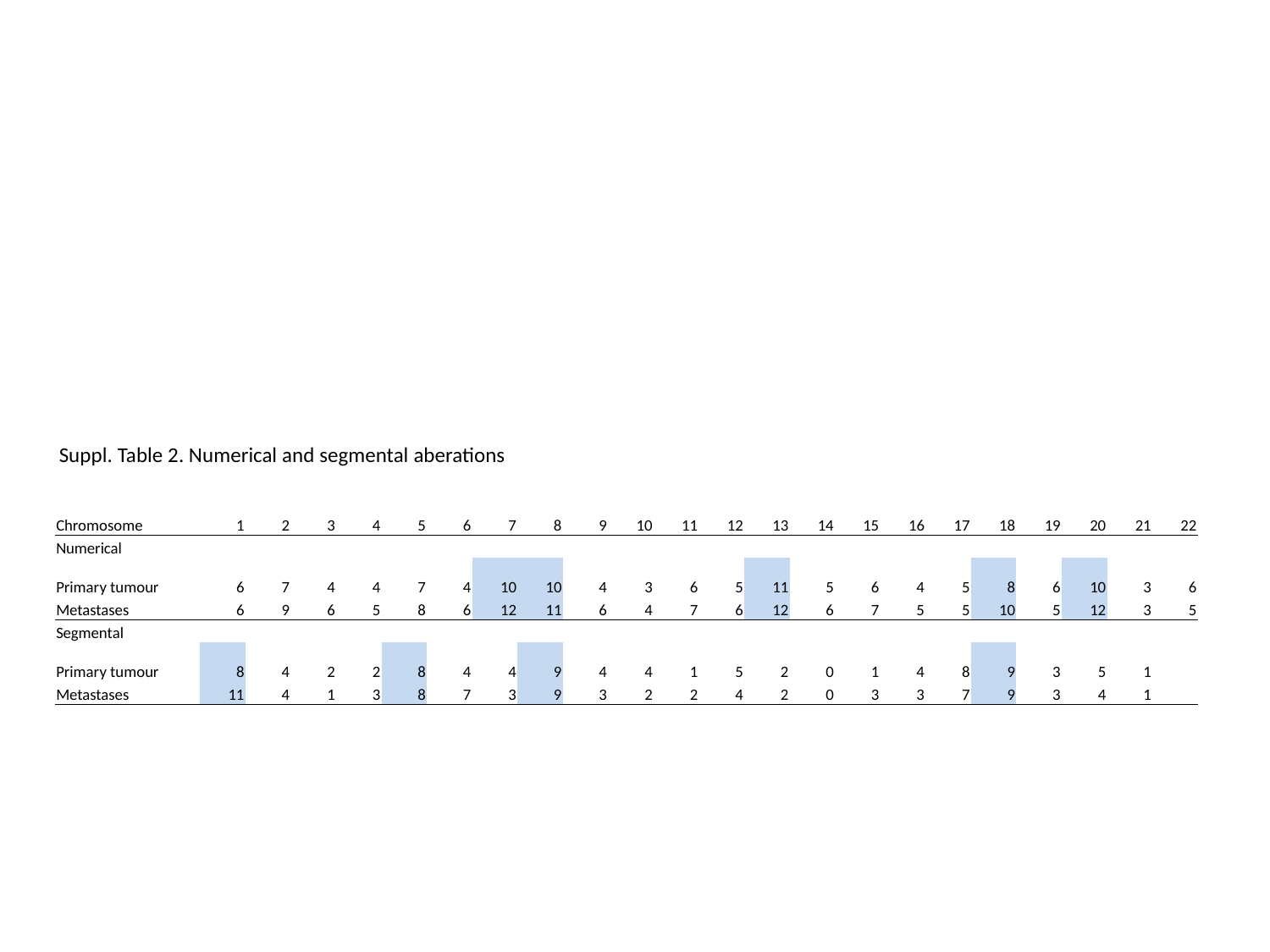

Suppl. Table 2. Numerical and segmental aberations
| Chromosome | 1 | 2 | 3 | 4 | 5 | 6 | 7 | 8 | 9 | 10 | 11 | 12 | 13 | 14 | 15 | 16 | 17 | 18 | 19 | 20 | 21 | 22 |
| --- | --- | --- | --- | --- | --- | --- | --- | --- | --- | --- | --- | --- | --- | --- | --- | --- | --- | --- | --- | --- | --- | --- |
| Numerical | | | | | | | | | | | | | | | | | | | | | | |
| Primary tumour | 6 | 7 | 4 | 4 | 7 | 4 | 10 | 10 | 4 | 3 | 6 | 5 | 11 | 5 | 6 | 4 | 5 | 8 | 6 | 10 | 3 | 6 |
| Metastases | 6 | 9 | 6 | 5 | 8 | 6 | 12 | 11 | 6 | 4 | 7 | 6 | 12 | 6 | 7 | 5 | 5 | 10 | 5 | 12 | 3 | 5 |
| Segmental | | | | | | | | | | | | | | | | | | | | | | |
| Primary tumour | 8 | 4 | 2 | 2 | 8 | 4 | 4 | 9 | 4 | 4 | 1 | 5 | 2 | 0 | 1 | 4 | 8 | 9 | 3 | 5 | 1 | |
| Metastases | 11 | 4 | 1 | 3 | 8 | 7 | 3 | 9 | 3 | 2 | 2 | 4 | 2 | 0 | 3 | 3 | 7 | 9 | 3 | 4 | 1 | |
