# Supplementary material for: Genomic alterations accompanying tumour evolution in colorectal cancer: tracking the differences between primary tumours and synchronous liver metastases by whole-exome sequencing
Source: BMC Cancer. 2018 Jul 20;18:752. doi: 10.1186/s12885-018-4639-4 (PMC6053835; doi:10.1186/s12885-018-4639-4)
Supplement: Supplementary file 4 — Table S4. Damaging private mutations (SIFT) identified to determine whether CNAs accompanied the mutation. (PPTX 47 kb) [file 12885_2018_4639_MOESM4_ESM.pptx]

## Slide 1
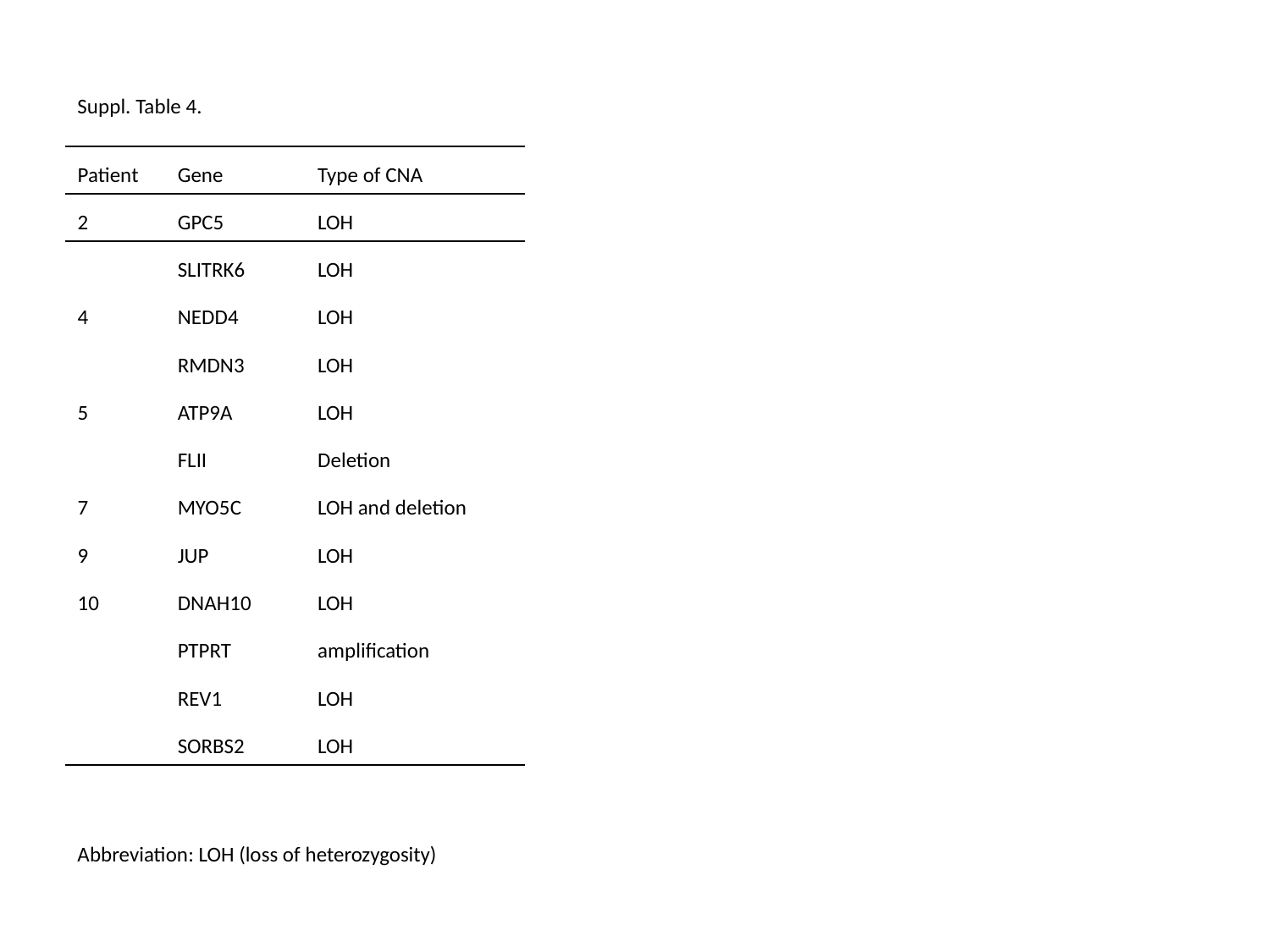

Suppl. Table 4.
| Patient | Gene | Type of CNA |
| --- | --- | --- |
| 2 | GPC5 | LOH |
| | SLITRK6 | LOH |
| 4 | NEDD4 | LOH |
| | RMDN3 | LOH |
| 5 | ATP9A | LOH |
| | FLII | Deletion |
| 7 | MYO5C | LOH and deletion |
| 9 | JUP | LOH |
| 10 | DNAH10 | LOH |
| | PTPRT | amplification |
| | REV1 | LOH |
| | SORBS2 | LOH |
Abbreviation: LOH (loss of heterozygosity)
